# Day-Ahead Crude Oil Price Forecasting Using a Novel Morphological Component Analysis Based Model

**DOI:** 10.1155/2014/341734

**Published:** 2014-06-25

**Authors:** Qing Zhu, Kaijian He, Yingchao Zou, Kin Keung Lai

**Affiliations:** ^1^School of Economics and Finance, Xi'an Jiaotong University, Xi'an 710049, China; ^2^International Business School, Shaanxi Normal University, Xi'an 710062, China; ^3^School of Economics and Management, Beijing University of Chemical Technology, Beijing 100029, China; ^4^College of Information Science and Technology, Beijing University of Chemical Technology, Beijing 100029, China; ^5^Department of Management Sciences, City University of Hong Kong, Tat Chee Avenue, Kowloon Tong, Kowloon, Hong Kong

## Abstract

As a typical nonlinear and dynamic system, the crude oil price movement is difficult to predict and its accurate forecasting remains the subject of intense research activity. Recent empirical evidence suggests that the multiscale data characteristics in the price movement are another important stylized fact. The incorporation of mixture of data characteristics in the time scale domain during the modelling process can lead to significant performance improvement. This paper proposes a novel morphological component analysis based hybrid methodology for modeling the multiscale heterogeneous characteristics of the price movement in the crude oil markets. Empirical studies in two representative benchmark crude oil markets reveal the existence of multiscale heterogeneous microdata structure. The significant performance improvement of the proposed algorithm incorporating the heterogeneous data characteristics, against benchmark random walk, ARMA, and SVR models, is also attributed to the innovative methodology proposed to incorporate this important stylized fact during the modelling process. Meanwhile, work in this paper offers additional insights into the heterogeneous market microstructure with economic viable interpretations.

## 1. Introduction

With the technological advancement and development of global economic integration, both the demand and the supply of crude oil are influenced by increasingly complex and diverse market participants around the world. This, together with numerous influential factors, such as weather conditions, political stability, economic prospects, consumer expectations, and business indicators, has led to the fluctuating price movement in the crude oil market. This situation has exacerbated in recent years accompanying the wave of liberalization and globalization, which are beyond the explanatory abilities provided by the current models and methodologies. Meanwhile, since crude oil is traded less frequently than equities, this results in higher levels of market imperfection and relatively lower levels of market efficiency, which leads to theoretically valuable and challenging research problems. Therefore, there has been arising interest from both academics and industries for more accurate modeling and forecasting of its price movement [1, 2].

Traditionally the structural and econometric models, mostly linear in nature, have been the mainstream approach in the crude oil forecasting field. ARMA model represents the typical time series approach while regression model as well as vector autoregressive (VAR) model represents the typical multivariate approaches. Mostly they offer satisfactory performance over the medium to long time horizon but fail over the short time horizons. This indicates that the characteristics of prices in the crude oil market contain unknown nonlinear features in the case of time series models, as well as nonlinear interrelations with other macroeconomic factors in the case of multivariate models. Current approaches alone can only offer insufficient level of explanatory and forecasting power for the price movement.

The nonparametric nonlinear artificial intelligence based approaches such as neural network, support vector regression, and empirical mode decomposition largely rely on the data mining exercises to extract nonlinear data patterns [3, 4]. They have shown some promising performance improvement. However, the performance improvement is not consistent for all test cases [5]. Meanwhile, arguments often arise for their results as they risk overfitting the data. Results solely that relied on these approaches also suffer from their powerful but “black box” approaches as limited insights into the underlying influencing factors with economic rationale can be inferred [3, 4, 6]. Therefore, better understanding of the underlying DGP and accurate forecasting in the crude oil market remains one of the most difficult problems in the field [1, 2].

Recent empirical researches have increasingly revealed the significance of multiscale data behaviors, where the mainstream approaches had failed to explain and incorporate during the modeling process. Therefore, the semiparametric paradigm based on computational harmonic analysis has emerged as the preferred alternative method [7]. This is based on the notion that the seemingly unstationary nonlinear data consist of different stationary data of both linear and nonlinear characteristics. This is consistent with HMH; that is, the market is heterogeneous in nature, and exploitation of this stylized fact leads to better understanding of the underlying DGPs and forecasting accuracy. Thus it is inappropriate to abandon the use of linear models in favor of nonlinear models, which could lead to ignorance of important deterministic patterns. However, different underlying DGPs also need to be incorporated during the modeling process. Linear models, however, capture only parts of them. For example, there are ample empirical evidences suggesting the transient data features, which lead to the extreme value theory in the forecasting field.

Thus, the multiscale decomposition method provides an important alternative but only a handful of works have explored it in this field. Some preliminary findings using the wavelet analysis to analyze the multiscale structure of DGP have led to some positive performance improvement in areas such as crude oil, electricity, equities, and exchange rate markets. These empirical studies have used one single family of wavelets to extract data features of interest. For example, wavelet analysis has been widely used to preprocess or denoise the data. For derivatives market, Haven et al. [8] and Almeida and Moriconi [9] use the wavelet analysis to denoise the option data and find that it significantly improves the option valuation accuracy [8, 9]. For crude oil market, Jammazi and Aloui [10] use wavelet analysis to denoise the data while shifting the focus of the paper on the choice of different activation functions on the performance improvement [10]. Yousefi et al. [11] use the wavelet analysis to decompose the crude oil price and extend the decomposed data components directly to make forecasts [11]. For electricity forecasting purpose, Amjady and Keynia [12], Meng et al. [13], and Aggarwal et al. [14] based their neural network forecasting model on the wavelet preprocessed data series and have obtained positive performance improvement [12–14]. Xu et al. [15] have attempted to combine the wavelet analysis with the support vector machine and have obtained positive performance improvement in the empirical studies in Australian electricity market [15]. Conejo et al. [16] have combined the wavelet analysis and ARMA model to analyze the Spanish electricity market and obtained positive results [16]. Meanwhile, another emerging trend is to identify the causal relationship or interrelationship among different financial markets. For example, recently, Benhmad [17] has conducted the empirical studies to test for Granger causality between oil price and US GDP and finds it varying across time scale domain [17]. Work in Reboredo and Rivera-Castro [18] and Reboredo and Rivera-Castro [19] has identified the contagion and interdependence relationship between crude oil and exchange rate, as well as the crude oil and the stock markets in Europe, respectively [18, 19].

As the real data naturally have multiple representations and are redundant when using a group of wavelet families to represent them, the single basis approach implies obviously very strong and questionable assumptions. Thus it is of critical importance to represent the DGP by using mixture of different bases, as suggested by recent empirical researches [7, 20, 21]. Since data now have multiple representations in the dictionary of bases with no unique solution, sparsity is proposed as the measure to guide the searching process for the optimal multiple bases representations. The true underlying component should concentrate on its band of influences, which usually represent a few significant points, which in turn correspond to the sparsity definition. Morphological component analysis (MCA) is one emerging technique in the field of sparsity representations of signals. Some positive performance improvements have been witnessed with limited applications in engineering fields where MCA has been used to extract multiple data features of interests [22–25]. For example, in image processing, Liang et al. [26] use the MCA to solve the face hallucination problem, whose resolution relies on the accurate decomposition of the original image into the global high resolution image and an unsharp mask [26]. Grosdidier and Baussard [27] use MCA to extract the target signature from the Range-Doppler images, which contributes to more accurate suppression of noises [27]. Abrial et al. [25] show that MCA can be applied to analysis of spherical data maps [25]. In the field of medical engineering, Gao et al. [28] propose MCA as an effective mammographic mass detection tool that could achieve satisfactory detection performance [28]. Meanwhile, applications of MCA also extend to different fields of engineering. Bobin et al. [29] extend MCA to multichannel case to solve the multichannel inverse problems [29]. Zeng et al. [30] use MCA to separate transient events and stationary noises based on their different morphological characteristics in vibration signals in the Watt experiment [30].

This paper proposes a morphological component analysis based hybrid methodology for modeling and forecasting of crude oil price. Results in this study explore and unveil the complex market structure consisting of data components of different data characteristics modeled using morphological component analysis. Empirical studies have been conducted in the marker benchmark West Taxes Intermediate (WTI) and Brent markets to investigate the performance improvements of the proposed model, against traditional benchmark models. The main contribution of this paper is the introduction of multiple basis based approach, to recover the underlying constituent component with only the prior information of the signals available to study the heterogeneous market structure without the inside information. This represents the important divergence from the widespread oversimplified single basis approach, inconsistent with market structure, and is only valid at macroscale. The introduction of MCA based approach incorporated the stylized fact that there are redundant forms of representations on the underlying data generating process, which need to be optimized, and contributes to the understanding and forecasting of the evolutions in the market microstructure.

This paper is organized as follows.  Section 2 provides a brief account of the sparsity decomposition and MCA theories behind.  Section 3 proposes and illustrates MCA based hybrid methodologies and their numerical procedures. Experiment results for empirical studies are reported and analyzed in Section 4, based on which finalizing conclusions are drawn in Section 5.

## 2. Relevant Theories

### 2.1. Sparsity Decomposition and Morphological Component Analysis

Over the years, numerous bases have been developed to capture particular data features. Typical bases used include global oscillating discrete sine and cosine transform (DST and DCT) and locally oscillating discrete wavelet transform (DWT). With the availability of a large number of bases to construct an overly complete dictionary, guiding measures are needed to select and distinguish bases for specific data features to provide the most efficient representations. Sparsity is one such measure. Different basis may be more efficient in representing particular data features and thus provide sparse representation better than for other data features. Meanwhile, sparsity provides a measure for bases to discriminate against different data features [23–25]. Therefore, the goal of sparsity decomposition is to search for the appropriate representation of signals based on the morphological diversity criteria. It attempts to represent the signals using a dictionary of overcomplete redundant basis dictionary and model them as the linear combination of different morphologically diversified components [24]. Formally it is defined as follows.

Suppose *D* is an overcomplete dictionary constructed as the union of orthonormal bases as *D* = [*ξ*_1_,…, *ξ*_*D*_], from a collection of signal waveforms or atoms *δ*_1_,…, *δ*_*K*_. The projection coefficient *α* can be defined for mapping signal *y* ∈ *R* ^*n*^ into the bases domain as in the following:

(1) $$\begin{array}{c} y=\alpha_{i}D. \end{array}$$

The signal is sparse in dictionary *D* if most entries of projection coefficients *α* are zero and there exist only a few significant ones. This constraint also ensures an uncertainty principle that states that a component that is sparse in a particular basis *δ*_*k*_ is not sparse in a mutually incoherent basis *δ*_*i*_, *i* ≠ *k*. This group of basis pairs may include DCF and wavelets and wavelets and Dirac bases.

Suppose signal *y* can be represented by a linear combination of *k* components *S*_*k*_, *k* = 1,…*K*, where each component *S*_*k*_ is sparse in the corresponding unique bases in dictionary *δ*_*k*_. The goal of sparsity decomposition is to search for components obtained from orthogonal transform with basis functions and provides the optimal linear representation of a data series *y*. This problem is formulated as the linear optimization problem in the following for *a*_*k*_:

(2) min⁡a1,…,ak ∑k=1K||ak||0s.t.  y=∑k=1Kδkak,

where the function *l*_*o*_ norm refers to ||*x*||_*o*_, that is, the support of *x*, counting the number of nonzero components of vector *x*.

Since it is a typical NP-hard combinatorial discrete optimization problem, algorithms with relaxed condition or approximate accuracy have been developed over the years to reduce computational complexity. These include the greedy matching pursuit (MP) [31], basis pursuit (BP) and basis pursuit denoising (BPDN) [32], LARS [33], and MCA [34]. Compared to BP, MCA represents an alternative efficient approach based on iterative thresholding algorithm.

Different orthogonal bases have been developed over the years. Typical examples are the traditional discrete cosine function (DCF), wavelets, and so forth. DCF is a global invariance function, well researched in the literature. Wavelets are more complex continuous functions that have high energy concentration over short intervals of time, which is in direct contrast to the globally time invariant sinusoid functions used in more traditional spectrum analysis tools such as Fourier analysis [35]. Wavelets are defined as solutions to the two-scale difference equation as in the following [36]:

(3) $$\begin{array}{c} C_{\psi }\left(x\right)=\sqrt{2}\underset{k\in z}{\sum }h_{k}\psi \left(2x-k\right). \end{array}$$

Therefore, wavelet functions satisfy both admissibility and unit energy conditions that guarantee their time scale localization with zero vanishing moments.

There are different families (or types) of wavelets. Each is capable of adapting to and accentuating certain data characteristics. Typical wavelet families include Haar, Daubechies, and Coiflets, each with different characteristics such as support and vanishing moments [36].

### 2.2. Support Vector Regression (SVR)

Support vector regression (SVR) is an emerging machine learning theory. It adopts the structural risk minimization principle during the data training and learning process and models it as a convex optimization problem to balance between fitting accuracy and model generalizability. Thus it alleviates the overfitting and local minima issues with the traditional supervised learning algorithm, such as neural network models, which are based on the empirical risk minimization principle [37]. Support vector regression is the extension of the support vector machine theory in regression analysis [37]. This is achieved introduction of some loss function such as the most popular *ϵ*-insensitive loss function. Typically for a set of data points (*x*_1_, *y*_1_), (*x*_2_, *y*_2_),…, (*x*_*m*_, *y*_*m*_), where *x*_*i*_ ∈ *R* ^*n*^, *y*_*i*_ ∈ *R* ^*n*^, the linear regression problem is formulated as

(4) $$\begin{array}{c} f\left(x\right)=\omega \phi \left(x\right)+b,\;\;\phi :R^{n}⟶F,\;\;\omega \in F, \end{array}$$

where *ϕ*(*x*_*i*_) denotes the transformation in feature *F*. Given nonlinear data, kernel function *k*〈*x*_*i*_ · *x*〉 is used for *ϕ*(*x*_*i*_) to map nonlinear inputs into linear inputs in higher dimensions. *w* is the weight in feature space *F*.

Introducing the *ϵ* loss function, the regression problem is further transformed into the convex optimization problem formulated as in

(5) min⁡ R(ω,ξi,ξi∗)=12||ω||2+Ci∑i=1n(ξi+ξi∗)s.t. {yi−ϕ(ω,t)−b≤εi+ξiϕ(ω,t)+bi−yi≤εi+ξi∗ξi,ξi∗≥0         Ci≥0          },

where *ϵ* is the loss function that measures the forecast deviations allowed. Two slack variables *ξ*_*i*_, *ξ*_i_* are introduced to measure the size of positive and negative deviations. *C* is the penalty variable for empirical errors. *w* ^*T*^ *w* is the regularized term.

Applying Lagrangian and Karush-Kuhn-Tucker conditions, the dual problem of the original optimization problem is formulated and solved as in the following to reduce dimensionality and computational costs:

(6) max⁡ L=∑i=1ai−12∑i,jaiajyiyjxiTxjs.t. {∑iaiyi=0ai≥0 ∀i}.

As an emerging technique, application of SVR in forecasting literature has been growing in recent years. It is typically viewed as an improvement over the traditional neural network models to avoid the local minima issue. Some very recent development includes its variant named multiple input multiple output SVR. For example, Bao et al. [38], Bao et al. [39], and Xiong et al. [40] have used the MIMO SVR in predicting the time series multiple steps ahead. The application evaluation of the proposed algorithm using the physics time series and stock market data has confirmed the improved predictive accuracy in the stock market [38–40]. Cao and Tay [41] and Ince and Trafalis [42] find separately that the performance of SVR is better than the neural network model [41, 42]. In electricity market, Aggarwal et al. [43] obtain significant performance improvement over traditional models and neural network models [43]. Zhao et al. [44] use the SVR model to forecast price movement and variance and constructed the prediction interval, claiming the model to be better than the traditional GARCH approach [44]. The SVR model is also being applied in forecasting crude oil price and has achieved superior positive performance. Xie et al. [45] construct a support vector regression based time series forecasting model for crude oil market and found its performance superior to backpropagation neural network and ARMA model [45]. Despite the positive results reported in the literature, SVR suffers from the same problems as neural network; that is, its performance is also sensitive to the chosen parameters, especially the trade-off parameters *C*, reflecting human preference for the balance between overfitting and generalizability. Recent progress in using the metaheuristics approach to determine the parameters has led to significantly improved performance. For example, Li and Tan [46] and Bao et al. [47] have shown that the evolutionary algorithms such as PSO and memetic algorithm can be used to determine more optimal model specifications for SVR and result in improved performance [46, 47]. Tian et al. [48] have also provided an alternative multiple kernel based framework that emphasizes the inductive approach to determine the parameters in SVR tuning [48]. Although there are debates on the economics insights that SVR based models can offer, SVR model serves as a very good optimization model during the forecasting process, especially when its parameters are fine-tuned with some advanced techniques.

## 3. A Morphological Component Analysis (MCA) Based Hybrid Methodology

The proposed MCA based hybrid methodology follows the “divide and conquer” principle. The theoretical basis behind the proposed approach is the proposition of heterogeneous market hypothesis (HMH), relaxing the homogeneous and rational assumption of efficient market hypothesis (EMH) underlying the majority of mainstream models. The rationale is that EMH assumes homogeneous time horizon, frequency, and individual characteristics in the data, which provides the acceptable level of approximations over the medium to long term time horizon when the market structure is relatively simpler due to strict regulations and demand for the forecasting accuracy, are only at moderate level. However, over the shorter interval, there are market imperfections that enable price predictability. Meanwhile, with increasingly complex market structure due to deregulation and technological development, recent empirical evidence in the exchange rate and equity markets suggests that the heterogeneous nature of market is no longer ignorable and could be the key to explain and reconcile EMH and empirical stylized facts that suggest price predictability [49, 50]. Meanwhile, the heterogeneous market hypothesis (HMH) arises to complement the traditional efficient market hypothesis (EMH) [51–57]. The HMH proposes that the market consists of heterogeneous agents with heterogeneous investment strategies and investment time horizons. Compared to the homogeneous reaction to the news shocks in the EMH, HMH states that these agents or investors react to news shocks differently based on their own characteristics. On one hand, their investment time horizon and dealing frequency are diversely different. For example pensions funds and central banks tend to have low dealing frequency focusing on long time horizon while the market traders tend to have high dealing frequency with short time horizon. On the other hand, these agents or investors employ diversely different investment strategies or measures based on their own characteristics and focus. In the meantime, as recent harmonic analysis research suggests, appropriate recovery of the underlying morphologically diversified components based on sparsity decomposition is important to data trend modeling. The original data series with the mixture of linear and nonlinear data characteristics need to be decomposed into the underlying morphologically diversified DGP, whose distributional characteristics conform to the assumptions of mainstream econometric models. Technically to study the heterogeneous market structure without inside information, innovative algorithms such as MCA that can recover the underlying constituent component are needed since only the prior information of the signals is available.

The proposed MCA approach also represents a significant paradigm shift from traditional approaches, over simplifying assumptions that are inconsistent with market structure, and is only valid at macroscale. The MCA based approach incorporates the stylized fact that there are redundant forms of signal representations. The accuracy of optimal extraction of components with overcomplete dictionary of bases that represent our assumptions has a limit, governed by the uncertainty principle. However, the approximation of accuracy can improve continuously with the development of technology. Based on HMH, MCA assumes that the data are influenced by some underlying components, which have morphologically diverse features, distinguishable with a dictionary of bases, together with additive noises as defined as in

(7) $$\begin{array}{c} r\left(t\right)=\sum \xi^{\left(i\right)}\alpha^{\left(i\right)}+n, \end{array}$$

where *r*(*t*) and *α* ^(*i*)^ refer to the original data series and underlying component series of morphologically different characteristics such as permanent and transient data characteristics. Consider *i* ∈ *k* where *δ*_1_,…, *δ*_*k*_ refers to the collection of morphologically different dictionaries including undecimated discrete wavelet, discrete cosine transform, and the Dirac basis. *ξ* ^(*i*)^ refers to the coefficient vectors for different complete dictionaries. *n* refers to the contaminating noises, possibly of Gaussian white noise of irrelevant nature.

MCA involves several key steps, including components extraction, feature identification, data modeling, and final forecast phases, as illustrated in Algorithm 1.

Firstly in the components extraction phase, the set of dictionaries with mutually incoherent bases is constructed. This is to ensure that the sparest solution can be found as the uncertainty principle ensures that no signal can have sparse representations in mutually incoherent bases simultaneously. Then, the underlying components can be extracted using the constructed dictionary based on MCA algorithm. The process of feature extraction in MCA follows the standard iterative thresholding algorithm as in Algorithm 2 [25, 34].

Secondly the nonlinear statistics test is used to test and identify linear and nonlinear data characteristics.

Thirdly appropriate model specifications from a set of model pools, consisting of linear, nonlinear, and random walk, are chosen and used to model the extracted components. Individual forecasts are made for each component accordingly. The optimal one is chosen based on the trial and error method.

For data components of linear nature, that is, if the price series are serially linearly dependent and not independent and identically distributed (i.i.d), the autoregressive moving average model (ARMA) is used to model the linear serial dependence in data, in which the current price level is linearly related to the past price level, incorporating the errors in previous forecasts as well. Typical ARMA model specification is estimated and forecasts are made as in

(8) $$\begin{array}{c} \hat{r_{t}}=\delta_{t}+\overset{m}{\underset{i=1}{\sum }}\phi_{i}r_{\left(t-i\right)}+\overset{n}{\underset{j=1}{\sum }}\theta_{j}\varepsilon_{\left(t-j\right)}+a_{t}, \end{array}$$

where *r*_*t*_ is the conditional mean of the data at time *t*, *r*_(*t*−*i*)_ is the lag *m* returns with parameter *ϕ*_*i*_, and *ε*_(*t*−*j*)_ is the lag *n* residuals in the previous period with parameter *θ*_*j*_. *δ*_*t*_ is the constant coefficient. *a*_*t*_ is the error term at time *t*.

For nonlinear data, the nonlinear model specification is estimated and forecasts are made as in

(9) $$\begin{array}{c} \hat{r_{t}}=\overset{p}{\underset{i=1}{\sum }}\omega_{i}r_{\left(t-i\right)}+b_{t}. \end{array}$$

If market is efficient, the random walk model is valid in that all past information is reflected in the current price, which is the only needed information to forecast the future movement. The price series is i.i.d and not predictable based on past information. Thus the random walk model remains a very important benchmark model as predictions from most linear models are less robust and are biased due to inappropriate extraction of patterns.

Based on the basic assumption in MCA analysis as in (7), the final forecasts are simply linear summation of individual forecasts made for different individual components as in

(10) $$\begin{array}{c} \hat{r(t)}=\hat{r_{\text{PT}}(t)}+\hat{r_{\text{TF}}(t)}. \end{array}$$

## 4. Empirical Studies

### 4.1. Data and Descriptive Statistics

As two representative benchmark marker markets were considered by US Energy Information Administration, both the US West Taxes Intermediate (WTI) crude oil market and the European Brent (Brent) are used as the testing fields for empirical studies in this paper. The experiments are designed following the convention in the literature. For both datasets, the performance evaluations of different models are conducted covering the time period from January 2, 2002, to February 13, 2009, when the latest event and data are incorporated while the impact of previous direct market disruptions, such as the Gulf war, is reduced to the minimum. This includes 1790 daily observations for WTI dataset and 1868 daily observations for Brent dataset. The data source is Energy Information Administration (EIA) of US Department of Energy. The datasets are conventionally divided on the 60–40 basis, which ensures sufficient samples for statistical significance of results. The first 60% of the dataset serves as the training set for estimating model specifications and parameters. The remaining 40% of dataset is reserved as the test set for evaluating performance of different models [58, 59]. The directional predictive accuracy of different models is evaluated using Pesaran-Timmermann test of directional predictive accuracy [60]. The original daily price series is log differenced at the first order to remove trend factors. The statistical predictive accuracy of different models is evaluated using mean squared error (MSE), for measuring the deviation of the predictive values from the actual observations, and the Clark-West statistical predictive accuracy test for nested models, for measuring the predictive accuracy between two nested models [61, 62]. The directional predictive accuracy of different models is evaluated using Pesaran-Timmermann test of directional predictive accuracy [60].

The calculated mean, standard deviation, skewness, and kurtosis for both WTI and Brent markets are 0.0011, 0.0230, −0.4326, and 4.8282 and 0.0011, 0.0218, −0.0763, and 4.3898, respectively. This suggests that on the aggregated level, crude oil market is relatively efficient and roughly normally distributed. However, the Jarque-Bera test of normality is rejected while the BDS test of independence is accepted at a low confidence level, that is, 70.8% in Brent market, which indicates that crude oil data deviate from normal and independent distribution. Therefore, this paper uses MCA techniques to extract morphologically distinct data components. In this paper, three different bases have been attempted, including DCT, Daubechies 4, and Symlet 4 wavelet family, where the number 4 is the order for the wavelet family.

The DCT and DST fail to extract the deterministic underlying components, which suggests the absence of long term periodic deterministic trends in the market. This is an interesting and provoking observation as it suggests that the market is characterized by salient data features and violation of the stationary character over long term in traditional modeling technique based on single time framework.

For components extracted using Daubechies 4 and Symlet 4, in WTI market, the calculated mean, standard deviation, skewness, and kurtosis are 0, 0.0041, −11.3492, and 273.8995 and 0, 0.0001, 0, and 1.5076, respectively. In Brent market, the calculated mean, standard deviation, skewness, and kurtosis are 0, 0.0022, 15.2061, and 329.3004 and 0, 0.0001, 0, and 1.5015, respectively. Thus distributions of all components extracted are significantly different from the normal distribution. This is also confirmed by the rejection of the Jarque-Bera test and BDS tests.

### 4.2. Experiment Results

Following conventions in the literature, three benchmark models, random walk (RW), ARMA, and SVR model, are used in the model evaluation process [63]. The lag order for benchmark ARMA(r,m) during the forecasting process is set to ARMA(1,1). The lag order p for benchmark SVR model is set to 2. The lag order for ARMA(r,m) in the forecasting process is determined based on the Akaike information criteria (AIC) and Bayesian information criteria (BIC) minimization principle.

Since very little guidance is available in the extant literature, parameters for MCA are determined using trial and error method. The rolling window is set to 512 to cover the relevant information set. Wavelet families used in the MCA analysis process include Daubechies 4 and Symlets 4 for WTI market, as well as Daubechies 6 and Symlets 6 for WTI market.

The *ϵ*-insensitive SVR is chosen with the radial basis function (RBF) kernel. The parameters for SVR model, including cost, gamma, epsilon, and tolerance of termination, are determined using the standard grid search method. They are listed in Table 1.

Predictive accuracy of the proposed algorithm against alternative benchmark models is as in Table 2.

Experiment results in Table 2 show the superior performance of the proposed MCA hybrid methodology, which outperforms individual benchmark models, random walk (RW), ARMA, and SVR model, in terms of predictive accuracy measured by MSE. Meanwhile, result of the Clark-West test of equal predicative accuracy suggests that the superior performance against benchmark models is statistically significant. The performance superiority is significant at 95% confidence level for WTI market and at 90% for Brent market.

Directional predictive accuracy of the proposed model against benchmark alternatives is listed in Table 3.

Experiment results in Table 3 show that the proposed MCA based methodology achieves a higher level of directional forecasting accuracy than the individual benchmark models, ARMA and SVR model, in terms of the ratio of correct predictions. Meanwhile, Pesaran-Timmermann test of directional predictive accuracy suggests that the superior performance against benchmark models is statistically significant. The performance superiority is significant at 90% confidence level for WTI market and at 95% for Brent market.

The optimal performance for WTI market is achieved when components extracted using Daubechies wavelet are modeled by the Random Walk model, while components extracted using Symlets wavelets are modeled by the SVR model. Interestingly the optimal performance for Brent market is achieved when components extracted using Daubechies wavelet are modeled by ARMA model while components extracted by using Symlets are modeled by the SVR model.

Therefore, these observations support the argument that crude oil prices are complicated processes with a mixture of underlying DGPs of different natures. Their modeling and analysis are tricky issues which require detailed investigation of the underlying structure in the time scale domain. Thus appropriate recovery of the underlying structure is critical to further performance improvement during the modeling process. Meanwhile, experiment results also show that the proposed algorithm is generalizable to different datasets with the flexibility to model nonlinear dynamics characterized by mixture of data of time varying natures.

## 5. Conclusions

Based on the HMH, this paper proposes a hybrid modeling methodology to incorporate multiscale market structure information into the modeling process, which provides a view of the microstructure of the underlying DGP, besides finer modeling accuracy. The proposed algorithm introduces the MCA techniques to analyze the multiscale market structure. Empirical studies on two major benchmark crude oil markets in the world suggest their effectiveness in analyzing the heterogeneous market structure and demonstrate significant positive performance improvement, as a result.

The proposed methodology reveals that the sparsity decomposition based methodology using MCA offers more complete and accurate representation of data features than the more widely used single basis methodology. The conventional single basis methodology provides only a partial and twisted view of data features by imposing strict assumptions in the inappropriate time domain. However, the proposed algorithm can capture more accurately the underlying DGPs of diverse natures, in both linear and nonlinear domain, by incorporating both multitime scale information and the multiple bases frequency feature information during the modeling process. Original results reported in this paper also merit further research on constructing redundant basis transform to explore the underlying GDPs in the seemingly fractal and chaotic financial market, whose characteristics can be revealed more clearly only in appropriate time and frequency settings. Meanwhile, research results in this paper also pave the way for further research into two largely overlooked assumptions behind mainstream wavelet bases research: the selection of appropriate basis to represent economic and financial data and the atomic decomposition of the underlying data features. The performance of the proposed algorithm is sensitive to the introduced bases parameter.

## Figures and Tables

**Algorithm 1:**
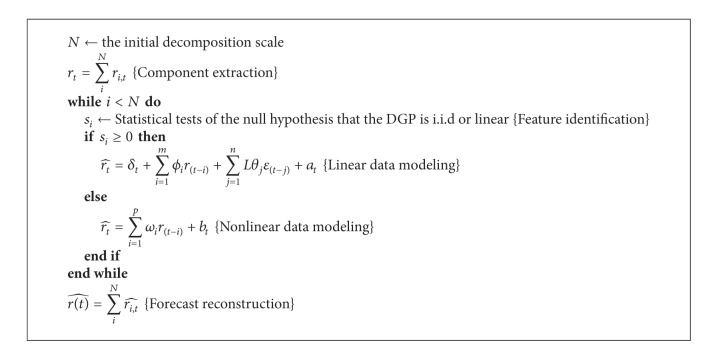
A MCA based hybrid methodology.

**Algorithm 2:**
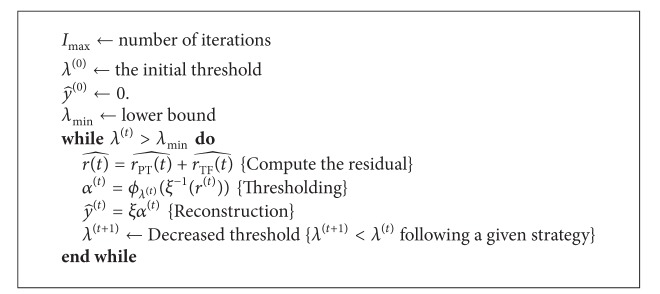
Iterative thresholding algorithm for MCA.

**Table 1:** Parameters chosen for SVR models.

Models	Cost	Gamma	Epsilon	Tolerance of termination
Original_WTI_	32	1	0.01562500	0.00050000
Daubechies 4_WTI_	8	0.06250000	0.06250000	0.00000010
Symmlet 4_WTI_	8	0.06250000	0.06250000	0.00000010

Original_Brent_	2	0.00781250	0.03125000	0.00040000
Daubechies 6_Brent_	32	0.06250000	0.00390625	0.00000600
Symmlet 6_Brent_	8	0.06250000	0.06250000	0.00000001

**Table 2:** Performance comparison of different models.

Models	MSE_×10^−4^_	CW__*P* value_^ARMA^_	CW__*P* value_^RW^_
RW_WTI_	8.8140	N/A	0.6641
ARMA_WTI_	8.8739	0.6641	N/A
SVR_WTI_	13.4749	0.5076	0.4304
MCA-RW-SVR_WTI_	8.7337	0.0077	0.0274

RW_Brent_	7.2035	N/A	0.4485
ARMA_Brent_	7.2153	0.4485	N/A
SVR_Brent_	12.0060	0.5838	0.6407
MCA-ARMA-SVR_Brent_	7.1106	0.0702	0.0568

**Table 3:** Pesaran-Timmermann directional test.

Models	Dstat_%_	PT
ARMA_WTI_	51.96	0.1227
SVR_WTI_	48.74	0.7499
MCA-RW-SVR_WTI_	52.65	0.0768

ARMA_Brent_	50.48	0.4524
SVR_Brent_	46.52	0.9707
MCA-ARMA-SVR_Brent_	54.30	0.0102

## References

[1] Yang CW, Hwang MJ, Huang BN (2002). An analysis of factors affecting price volatility of the US oil market. *Energy Economics*.

[2] Plourde A, Watkins GC (1998). Crude oil prices between 1985 and 1994: how volatile in relation to other commodities?. *Resource and Energy Economics*.

[3] Yu L, Wang S, Lai KK (2008). Forecasting crude oil price with an EMD-based neural network ensemble learning paradigm. *Energy Economics*.

[4] Zhang X, Lai KK, Wang S-Y (2008). A new approach for crude oil price analysis based on empirical mode decomposition. *Energy Economics*.

[5] Clements MP, Franses PH, Swanson NR (2004). Forecasting economic and financial time-series with non-linear models. *International Journal of Forecasting*.

[6] He K, Lai KK, Guu SM, Zhang J (2008). A wavelet based multi scale var model for agricultural market. *Computation and Optimization in Information Systems and Management Sciences*.

[7] He K, Xie C, Lai K (2008). Estimation of value-at-risk for exchange risk via kernel based nonlinear ensembled multi scale model. *Advances in Neural Networks—ISNN 2008*.

[8] Haven E, Liu X, Shen L (2012). De-noising option prices with the wavelet method. *European Journal of Operational Research*.

[9] de Almeida VTX, Moriconi L (2012). Option pricing from wavelet-filtered financial series. *Physica A: Statistical Mechanics and Its Applications*.

[10] Jammazi R, Aloui C (2012). Crude oil price forecasting: experimental evidence from wavelet decomposition and neural network modeling. *Energy Economics*.

[11] Yousefi S, Weinreich I, Reinarz D (2005). Wavelet-based prediction of oil prices. *Chaos, Solitons and Fractals*.

[12] Amjady N, Keynia F (2008). Day ahead price forecasting of electricity markets by a mixed data model and hybrid forecast method. *International Journal of Electrical Power and Energy Systems*.

[13] Meng K, Dong ZY, Wong KP (2009). Self-adaptive radial basis function neural network for short-term electricity price forecasting. *IET Generation, Transmission and Distribution*.

[14] Aggarwal SK, Saini LM, Kumar A (2008). Electricity price forecasting in ontario electricity market using wavelet transform in artificial neural network based model. *International Journal of Control, Automation and Systems*.

[15] Xu Z, Dong Z, Liu W (2003). Short-term electricity price forecasting using wavelet and svm techniques. *Dynamics of Continuous Discrete and Impulsive Systems B: Applications & Algorithms*.

[16] Conejo AJ, Plazas MA, Espínola R, Molina AB (2005). Day-ahead electricity price forecasting using the wavelet transform and ARIMA models. *IEEE Transactions on Power Systems*.

[17] Benhmad F (2012). Modeling nonlinear Granger causality between the oil price and U.S. dollar: a wavelet based approach. *Economic Modelling*.

[18] Reboredo JC, Rivera-Castro MA (2013). A wavelet decomposition approach to crude oil price and exchange rate dependence. *Economic Modelling*.

[19] Reboredo JC, Rivera-Castro MA (2014). Wavelet-based evidence of the impact of oil prices on stock returns. *International Review of Economics and Finance*.

[20] He K, Xie C, Lai KK A wavelet denoising support vector regression ensemble model for exchange rate prediction.

[21] Kaijian H, Chi X, Kin KL Multi scale nonlinear ensemble model for foreign exchange rate prediction.

[22] Fadili JM, Starck J-L, Elad M, Donoho DL (2010). MCALab: reproducible research in signal and image decomposition and inpainting. *Computing in Science and Engineering*.

[23] Bobin J, Starck J-L, Moudden Y, Fadili MJ (2008). Blind source separation: the sparsity revolution. *Advances in Imaging and Electron Physics*.

[24] Bobin J, Starck J-L, Fadili JM, Moudden Y, Donoho DL (2007). Morphological component analysis: an adaptive thresholding strategy. *IEEE Transactions on Image Processing*.

[25] Abrial P, Moudden Y, Starck J-L (2007). Morphological component analysis and inpainting on the sphere: application in physics and astrophysics. *Journal of Fourier Analysis and Applications*.

[26] Liang Y, Xie X, Lai J-H (2013). Face hallucination based on morphological component analysis. *Signal Processing*.

[27] Grosdidier S, Baussard A (2012). Ship detection based on morphological component analysis of high-frequency surface wave radar images. *IET Radar, Sonar and Navigation*.

[28] Gao X, Wang Y, Li X, Tao D (2010). On combining morphological component analysis and concentric morphology model for mammographic mass detection. *IEEE Transactions on Information Technology in Biomedicine*.

[29] Bobin J, Moudden Y, Fadili J, Starck J-L (2009). Morphological diversity and sparsity for multichannel data restoration. *Journal of Mathematical Imaging and Vision*.

[30] Zeng W, Jiang X, Smith IM, Scott P (2010). Transient signal separation in Watt balance experiments. *Physics Letters A: General, Atomic and Solid State Physics*.

[31] Mallat SG, Zhang Z (1993). Matching pursuits with time-frequency dictionaries. *IEEE Transactions on Signal Processing*.

[32] Chen SS, Donoho DL, Saunders MA (2001). Atomic decomposition by basis pursuit. *SIAM Review*.

[33] Efron B, Hastie T, Johnstone I (2004). Least angle regression. *Annals of Statistics*.

[34] Starck J-L, Elad M, Donoho D (2004). Redundant multiscale transforms and their application for morphological component separation. *Advances in Imaging and Electron Physics*.

[35] Gencay R, Selcuk F, Whitcher B (2002). *An Introduction to Wavelets and Other Filtering Methods in Finance and Economics*.

[36] Percival DB, Walden AT (2000). *Wavelet Methods for Time Series Analysis*.

[37] Vapnik VN (2000). *The Nature of Statistical Learning Theory*.

[38] Bao Y, Xiong T, Hu Z (2014). Multi-step-ahead time series prediction using multiple-output support vector regression. *Neurocomputing*.

[39] Bao Y, Xiong T, Hu Z (2014). Pso-mismo modeling strategy for multistep-ahead time series prediction. *IEEE Transactions on Cybernetics*.

[40] Xiong T, Bao Y, Hu Z (2014). Multiple-output support vector regression with a firefly algorithm for interval-valued stock price index forecasting. *Knowledge-Based Systems*.

[41] Cao L, Tay FEH (2001). Financial forecasting using support vector machines. *Neural Computing and Applications*.

[42] Ince H, Trafalis TB (2008). Short term forecasting with support vector machines and application to stock price prediction. *International Journal of General Systems*.

[43] Aggarwal SK, Saini LM, Kumar A (2009). Day-ahead price forecasting in ontario electricity market using variable-segmented support vector machine-based model. *Electric Power Components and Systems*.

[44] Zhao JH, Dong ZY, Xu Z, Wong KP (2008). A statistical approach for interval forecasting of the electricity price. *IEEE Transactions on Power Systems*.

[45] Xie W, Yu L, Xu S, Wang S (2006). A new method for crude oil price forecasting based on support vector machines. *Computational Science—ICCS 2006*.

[46] Li S, Tan M (2010). Tuning {SVM} parameters by using a hybrid clpsobfgs algorithm. *Neurocomputing*.

[47] Bao Y, Hu Z, Xiong T (2013). A PSO and pattern search based memetic algorithm for SVMs parameters optimization. *Neurocomputing*.

[48] Tian X, Gasso G, Canu S (2012). A multiple kernel framework for inductive semi-supervised SVM learning. *Neurocomputing*.

[49] Müller UA, Dacorogna MM, Davé RD, Olsen RB, Pictet OV, von Weizsäcker JE (1997). Volatilities of different time resolutions—analyzing the dynamics of market components. *Journal of Empirical Finance*.

[50] Lux T, Marchesi M (1999). Scaling and criticality in a stochastic multi-agent model of a financial market. *Nature*.

[51] Brock WA, Hommes CH (1997). A rational route to randomness. *Econometrica*.

[52] Brock WA, Kleidon AW (1992). Periodic market closure and trading volume. A model of intraday bids and asks. *Journal of Economic Dynamics and Control*.

[53] Brock WA, LeBaron BD (1996). A dynamic structural model for stock return volatility and trading volume. *Review of Economics and Statistics*.

[54] Hommes CH (2001). Financial markets as nonlinear adaptive evolutionary systems. *Quantitative Finance*.

[55] Dacorogna MM, Genay R, Muller UAO, Pictet RBVO (2001). *An Introduction to High-Frequency Finance*.

[56] Farmer JD (2002). Market force, ecology and evolution. *Industrial and Corporate Change*.

[57] Goeree JK, Hommes CH (2000). Heterogeneous beliefs and the non-linear cobweb model. *Journal of Economic Dynamics and Control*.

[58] Yu L, Wang S, Lai KK (2005). A novel nonlinear ensemble forecasting model incorporating GLAR and ANN for foreign exchange rates. *Computers and Operations Research*.

[59] Walczak S (2001). An empirical analysis of data requirements for financial forecasting with neural networks. *Journal of Management Information Systems*.

[60] Pesaran MH, Timmermann A (1992). A simple nonparametric test of predictive performance. *Journal of Business and Economic Statistics*.

[61] Clark TE, West KD (2006). Using out-of-sample mean squared prediction errors to test the martingale difference hypothesis. *Journal of Econometrics*.

[62] Clark TE, West KD (2007). Approximately normal tests for equal predictive accuracy in nested models. *Journal of Econometrics*.

[63] Meese RA, Rogoff K (1983). Empirical exchange rate models of the seventies. Do they fit out of sample?. *Journal of International Economics*.

